# A novel optical thermometry based on the energy transfer from charge transfer band to Eu^3+^-Dy^3+^ ions

**DOI:** 10.1038/s41598-017-06421-7

**Published:** 2017-07-20

**Authors:** Jing Wang, Yanyan Bu, Xiangfu Wang, Hyo Jin Seo

**Affiliations:** 1 Department of Physics and Interdisciplinary Program of Biomedical, Mechanical & Electrical Engineering, Pukyong National University, Busan, 608-737 Republic of Korea; 2 College of Electronic Science and Engineering, Nanjing University of Posts and Telecommunications, Nanjing, 210046 People’s Republic of China

## Abstract

Optical thermometry based on the up-conversion intensity ratio of thermally coupled levels of rare earth ions has been widely studied to achieve an inaccessible temperature measurement in submicron scale. In this work, a novel optical temperature sensing strategy based on the energy transfer from charge transfer bands of W-O and Eu-O to Eu^3+^-Dy^3+^ ions is proposed. A series of Eu^3+^/Dy^3+^ co-doped SrWO_4_ is synthesized by the conventional high-temperature solid-state method. It is found that the emission spectra, emission intensity ratio of Dy^3+^ (572 nm) and Eu^3+^ (615 nm), fluorescence color, lifetime decay curves of Dy^3+^ (572 nm) and Eu^3+^ (615 nm), and relative and absolute sensitivities of Eu^3+^/Dy^3+^ co-doped SrWO_4_ are temperature dependent under the 266 nm excitation in the temperature range from 11 K to 529 K. The emission intensity ratio of Dy^3+^ (572 nm) and Eu^3+^ (615 nm) ions exhibits exponentially relation to the temperature due to the different energy transfer from the charge transfer bands of W-O and Eu-O to Dy^3+^ and Eu^3+^ ions. In this host, the maximum relative sensitivity S_r_ can be reached at 1.71% K^−1^, being higher than those previously reported material. It opens a new route to obtain optical thermometry with high sensitivity through using down-conversion fluorescence under ultraviolet excitation.

## Introduction

Recently, white light emitting diode (LED) technology has attracted much attention in the solid-state lighting industry, due to the advantages of white LEDs including power saving, long lifetime, and environmental benefit^1–3^. Single-phase luminescent materials that can directly emit white light under UV excitation have been explored in oxyfluoride glass and oxides matrices^4, 5^. The Eu^3+^ and Dy^3+^ ions were chosen as the red, green, and blue emitting activator centers through the transitions ^5^D_0_ → ^7^F_2_ (Eu^3+^), ^4^F_9/2_ → ^6^H_13/2_ (Dy^3+^) and ^4^F_9/2_ → ^6^H_15/2_ (Dy^3+^) under UV excitation^6–8^. For examples, Das and co-authors reported the controllable white light emission from Dy^3+^-Eu^3+^ co-doped KCaBO_3_ phosphor^6^. Laguna reported the shape controlled white light emission from Dy^3+^-Eu^3+^ co-doped CaMoO_4_ microarchitectures^7^. Hirai obtained the white light emission from Dy^3+^-Eu^3+^ co-doped Sr_2_CeO_4_^8^. In these works, the white light emission was controlled by changing doping concentration and host types.

It was reported that the temperature was a key parameter to adjust the emission intensity, the fluorescence intensity ratio, and emission color^9–12^. Berry found that the lifetime of ^5^D_0_ of Eu^3+^ ion was temperature dependent in Europium Tris(2,2,6,6-tetramethyl-3,5- heptanedionato)^9^. Morgan observed that the homogeneous linewidth of the ^5^D_0_ → ^7^F_0_ transition of Eu^3+^ was dependent on temperature in amorphous hosts^10^. Eckert observed that the phosphorescence decay lifetime of the Dy^3+^-transitions in Dy^3+^: Al_2_O_3_ showed strong temperature dependency in a temperature range from 1100 to 1500 K^11^. Zhou reported that the emission intensity ratio of ^5^D_1_ to ^5^D_0_ of Eu^3+^-doped transparent MF_2_ (M = Ba, Ca, Sr) glass ceramics increased with the temperature increase^12^. However, the temperature dependent optical property of Dy^3+^-Eu^3+^ co-doped materials has not been studied so far. It is necessary to explore the spectra and energy transfer of Dy^3+^-Eu^3+^ co-doped materials at high temperature.

From the published work on the spectra of Dy^3+^-Eu^3+^ co-doped materials, one can find that it had a little overlap of the excitation spectrum between ^5^D_0_ → ^7^F_2_ (Eu^3+^) and ^4^F_9/2_ → ^6^H_13/2_ (Dy^3+^)^13, 14^. It is necessary to find another ion to sensitize the Dy^3+^ and Eu^3+^ simultaneously. Notably, the charge transfer band of W-O was reported to have the wide absorption band in the ultraviolet range from 200 nm to 300 nm^15–17^. It may be a promise sensitizer to excite Dy^3+^ and Eu^3+^ simultaneously. Thus, in this work, the optical temperature property of Eu^3+^/Dy^3+^ co-doped SrWO_4_ are studied under 266 nm excitation. It is observed that the fluorescence intensity ratio between Eu^3+^ and Dy^3+^ emissions are strongly dependent on the temperature at the temperature range from 11 K to 529 K. The Eu^3+^/Dy^3+^ co-doped SrWO_4_ phosphors are proved as an excellent materials used for optical thermometry, due to its maximum value of S_r_ as high as 1.71% K^−1^.

## Results

**Figure 1:**
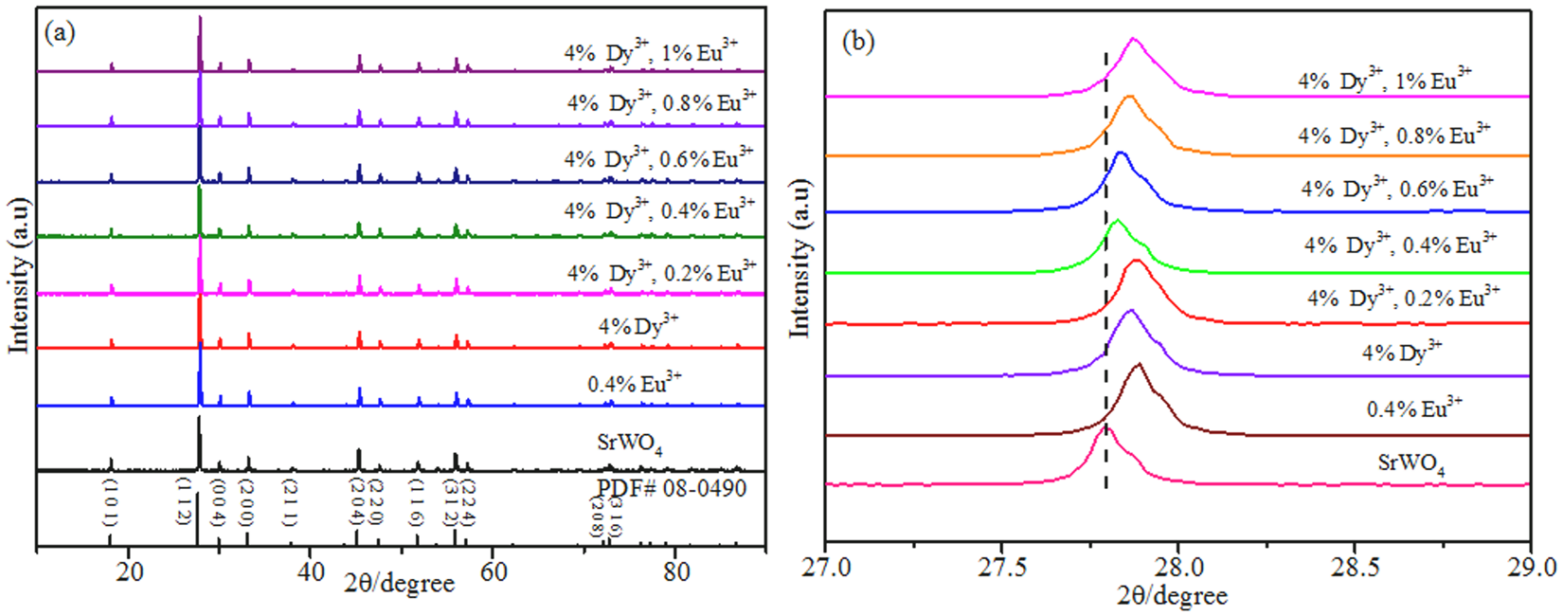
(**a**) XRD patterns of the as-synthesized SrWO_4_, SrWO_4_: 0.4 mol% Eu^3+^, and SrWO_4_: *x* Eu^3+^, 4 mol% Dy^3+^ (*x* = 0, 0.2 mol%, 0.4 mol%, 0.6 mol%, 0.8 mol%, 1 mol%) phosphors. The standard data of tetragonal SrWO_4_ (PDF# 08-0490) is given as a reference; (**b**) Partially enlarged XRD patterns of the corresponding phosphors (2θ = 27–29°).

**Figure 2:**
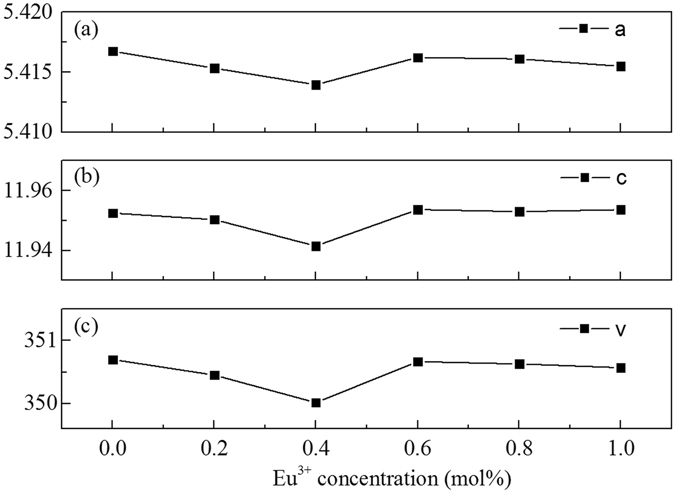
(**a**) Unit cell parameters of *a* (Å) and (**b**) *c* (Å) and (**c**) unit cell volume (Å^3^) at different Eu^3+^ concentration in tetragonal SrWO_4_: *x* Eu^3+^, 4 mol% Dy^3+^ (*x* = 0, 0.2 mol%, 0.4 mol%, 0.6 mol%, 0.8 mol%, 1 mol%).

The X-ray diffraction (XRD) patterns of the SrWO_4_, SrWO_4_: 0.4 mol% Eu^3+^, and SrWO_4_: *x* Eu^3+^, 4 mol% Dy^3+^ (*x* = 0, 0.2 mol%, 0.4 mol%, 0.6 mol%, 0.8 mol%, 1 mol%) samples synthesized by high-temperature solid-state reaction method are shown in Fig. 1. The peaks of all the products can be easily indexed to tetragonal system of SrWO_4_, which has a I41/a space group (PDF# 08-0490, unit cell parameters: a = b = 5.416 Å, c = 11.95 Å). No trace of impurity peaks can be found when Dy^3+^ and Eu^3+^ ions are introduced into the system. Compared with the pure SrWO_4_, the diffraction peaks of the Eu^3+^, Dy^3+^ single-doped and Eu^3+^/Dy^3+^co-doped SrWO_4_ exhibit a slight shift toward high-angle side, due to substitution of Sr^2+^ (1.26 Å, CN = 8) ions by smaller size Dy^3+^ (1.03 Å, CN = 8) and Eu^3+^ (1.07 Å, CN = 8) ions, which revealing that Dy^3+^ and Eu^3+^ ions have been successfully doped into the system^18, 19^. Figure 2 shows the unit cell parameters of *a* (Å) and *c* (Å) as well as unit cell volume (Å^3^). It can be observed that the value of lattice parameter *a* (Å) decreases firstly due to substitution of Sr^2+^ ions by smaller size Dy^3+^ and Eu^3+^ ions, and then increases with the increase of Eu^3+^ concentration due to the size differences between the different valence state cations^20, 21^. The same tendency can be observed in the values of parameter *c* (Å) and volume (Å^3^). It reveals that Eu^3+^ and Dy^3+^ ions can be easily doped into SrWO_4_ lattice, and the lattice can be distorted by the doping ions.

**Figure 3:**
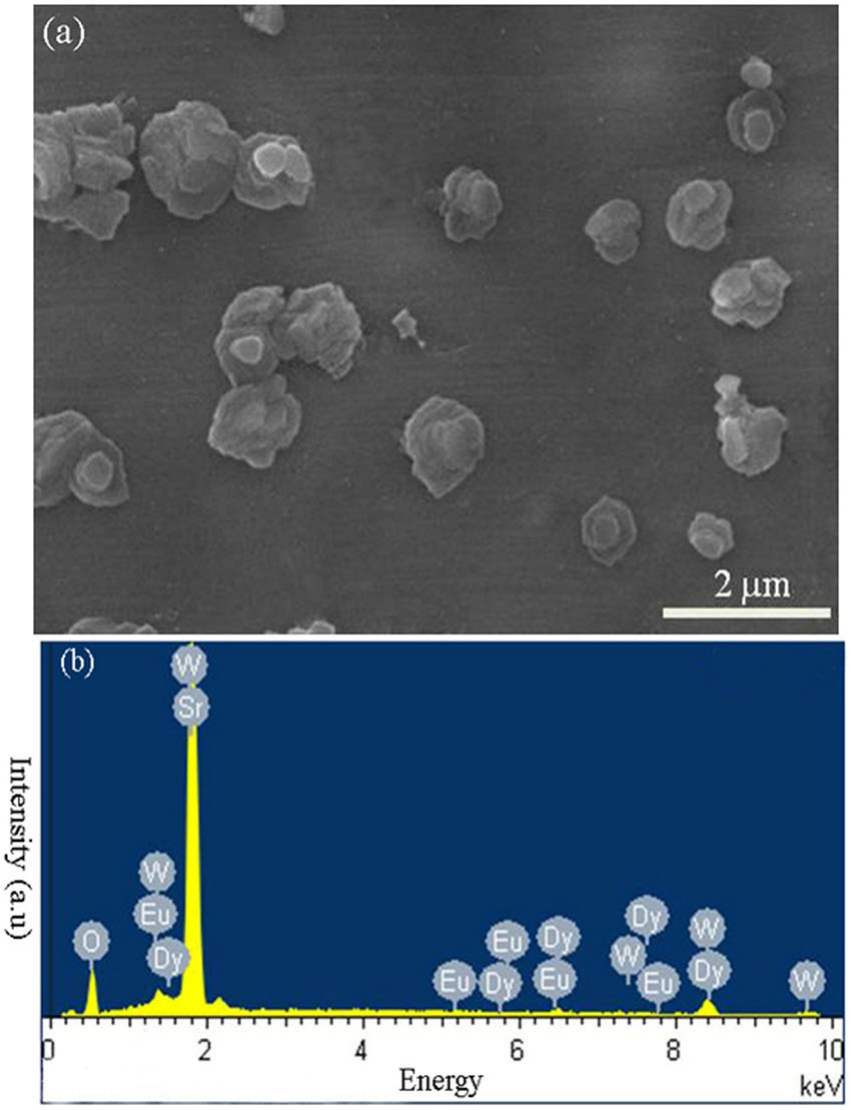
(**a**) SEM and (**b**) EDS images of the Eu^3+^/Dy^3+^ co-doped SrWO_4_ phosphor.

The scanning electron microscopy (SEM) image of a representative SrWO_4_: 0.4 mol% Eu^3+^, 4 mol% Dy^3+^ sample is shown in Fig. 3a, exhibiting sphere-like morphology with a particle size of about 1 µm. The energy dispersive spectrometer (EDS) spectrum (Fig. 3b) confirms the presence of Sr, W, O, Eu, and Dy elements, and further providing the evidence that Dy^3+^ and Eu^3+^ ions have been successfully doped into the SrWO_4_ host lattice.

**Figure 4:**
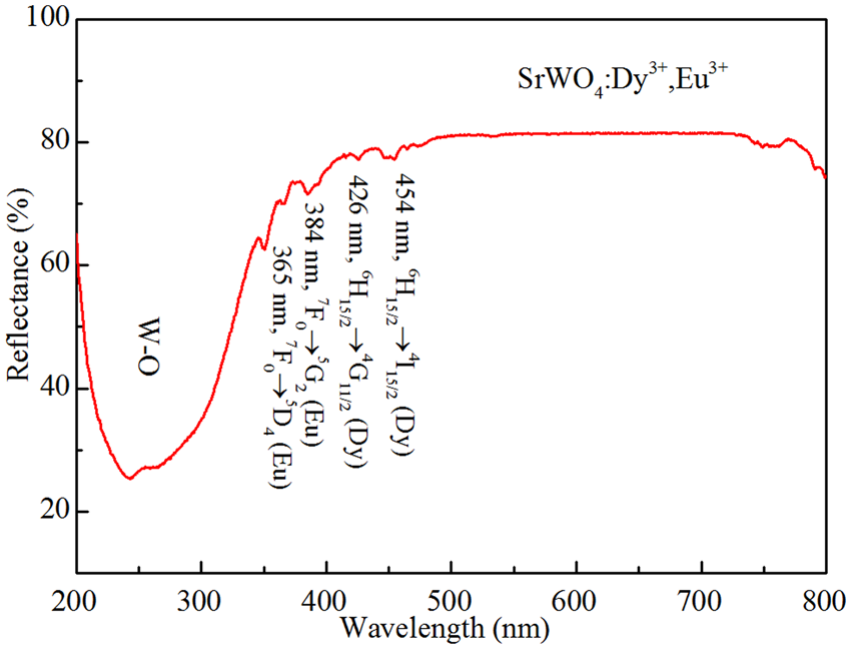
The ultraviolet-visible diffuse reflectance spectrum of the SrWO_4_: 0.4 mol% Eu^3+^, 4 mol% Dy^3+^ phosphor at room temperature.

The ultraviolet-visible diffuse reflectance spectrum of the SrWO_4_: 0.4 mol% Eu^3+^, 4 mol% Dy^3+^ in the range of 200–800 nm is shown in Fig. 4. A broad band and several absorption peaks corresponding to the doped ions can be observed. The broad band is located from 200 to 350 nm, corresponding to the O-W ligand-to-metal charge transfer in the WO_4_^2−^ group^22, 23^. Four absorption peaks located at 365, 384, 426 and 454 nm can be assigned to the intra 4 *f* electronic transitions of ^7^F_0_ → ^5^D_4_ (Eu^3+^), ^7^F_0_ → ^5^G_2_ (Eu^3+^), ^6^H_15/2_ → ^4^G_11/2_ (Dy^3+^), and ^6^H_15/2_ → ^4^I_15/2_ (Dy^3+^), respectively.

**Figure 5:**
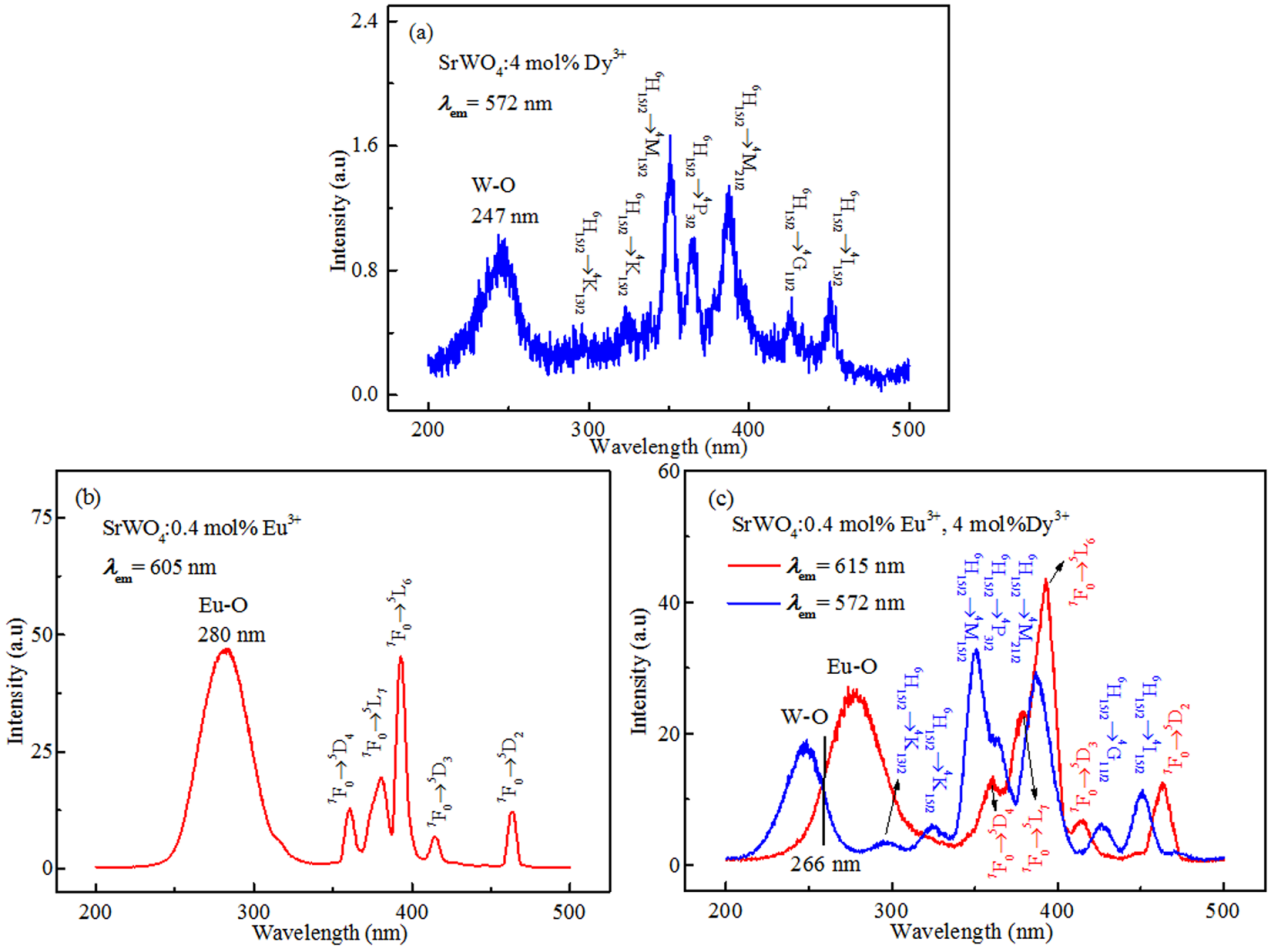
Excitation spectra of (**a**) SrWO_4_: 4 mol% Dy^3+^, (**b**) SrWO_4_: 0.4 mol% Eu^3+^, and (**c**) SrWO_4_: 0.4 mol% Eu^3+^, 4 mol% Dy^3+^ phosphors at room temperature.

The PLE spectra of SrWO_4_: 4 mol% Dy^3+^, SrWO_4_: 0.4 mol% Eu^3+^, and SrWO_4_: 0.4 mol% Eu^3+^, 4 mol% Dy^3+^ samples are shown in Fig. 5. The PLE spectrum of SrWO_4_: 4 mol% Dy^3+^ (Fig. 5a) illustrates a broad charge transfer band centered at 247 nm from 200 nm to 280 nm and a series of sharp lines extended to visible region can be observed by monitoring at 572 nm. The broad band can be ascribed to the charge transfer from WO_4_^2−^ group to Dy^3+^ ^24^, and the seven sharp lines can be ascribed to f–f transitions of Dy^3+^ 4 *f* configuration, which are ^6^H_15/2_ → ^4^K_13/2_ (296 nm), ^6^H_15/2_ → ^4^K_15/2_ (322 nm), ^6^H_15/2_ → ^4^M_15/2_ (350 nm), ^6^H_15/2_ → ^4^P_3/2_ (365 nm), ^6^H_15/2_ → ^4^M_21/2_ (387 nm), ^6^H_15/2_ → ^4^G_11/2_ (426 nm), and ^6^H_15/2_ → 4I_15/2_ (450 nm), respectively^25^. The excitation spectrum of SrWO_4_: 0.4 mol% Eu^3+^ is shown in Fig. 5b. Monitored at 615 nm, an intense broad band can be found in the range of 250–320 nm, which is due to the Eu-O charge transfer transition^24, 26^. While in the range of 200–250 nm, no obvious band can be found, indicating the energy transfer from WO_4_^2−^ group to Eu^3+^ is negligible. Additionally, a series of sharp lines corresponding to the intra 4 f electron transitions of Eu^3+^ ion can also be observed, which are 360 nm (^7^F_0_ → ^5^D_4_), 380 nm (^7^F_0_ → ^5^L_7_), 393 nm (^7^F_0_ → ^5^L_6_), 414 nm (^7^F_0_ → ^5^D_3_), and 463 nm (^7^F_0_ → ^5^D_2_), respectively^27^. Figure 5c shows the excitation spectra of SrWO_4_: 0.4 mol% Eu^3+^, 4 mol% Dy^3+^ phosphors. When compared with the excitation spectrum of SrWO_4_: 4 mol% Dy^3+^ by monitoring at 572 nm, the position of broad band and the excitation peaks in both the spectra can be matched well with each other. Nevertheless, the excitation intensity of Dy^3+^ is greatly enhanced when Eu^3+^ is introduced. When monitored at 615 nm, the Eu^3+^ excitation intensity decreases compared with the excitation spectrum of SrWO_4_: 0.4 mol% Eu^3+^. This may be due to the energy transfer from Eu^3+^ to Dy^3+^. The apparent overlap of charge transfer band centered at about 266 nm can also be observed. Hence, 266 nm pulsed laser is selected as the excitation light source to excite Dy^3+^ and Eu^3+^ ions.

**Figure 6:**
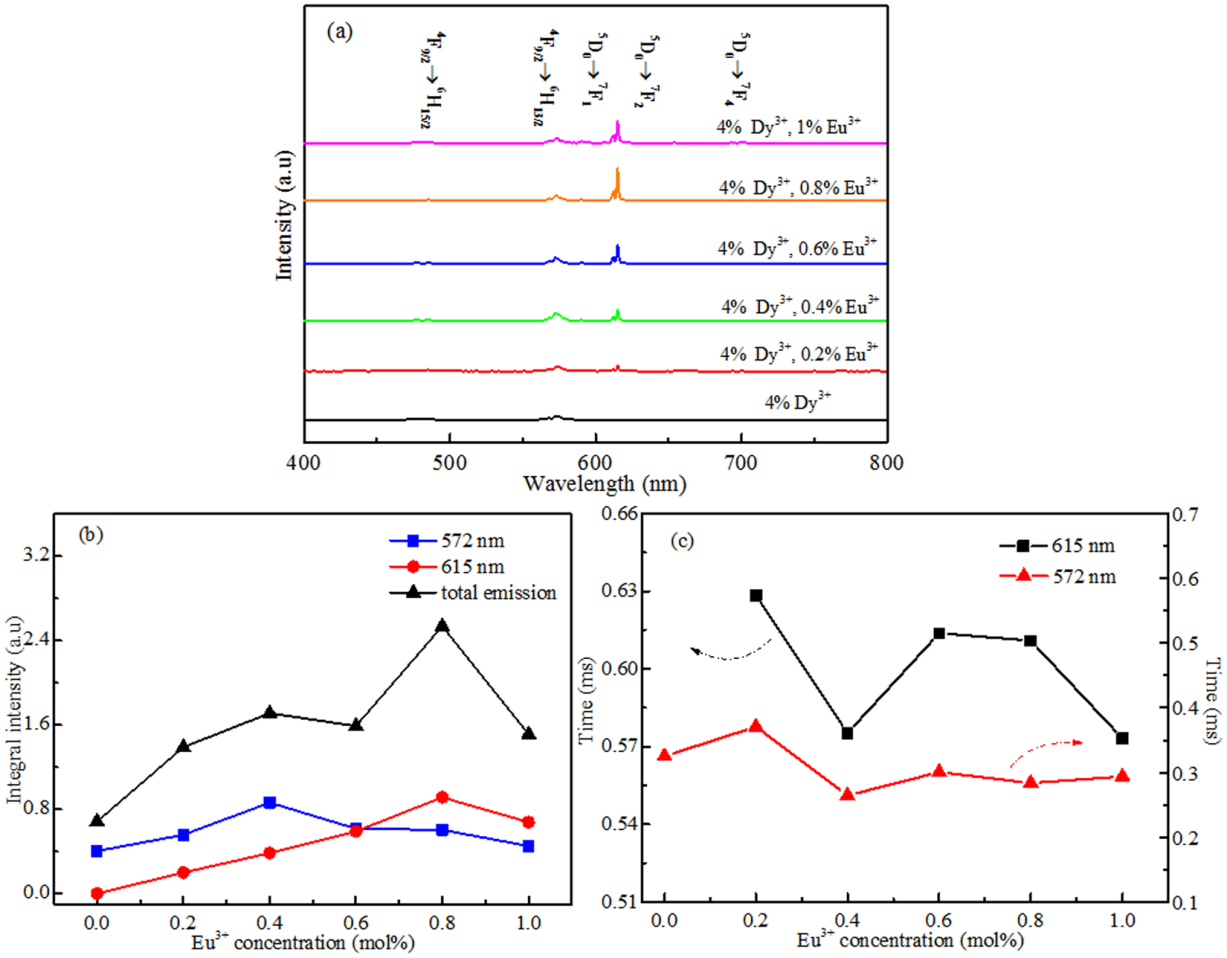
(**a**) PL emission spectra, (**b**) Integral intensity of Dy^3+^ (572 nm), Eu^3+^ (615 nm) and total emission, and (**c**) Calculated lifetimes of ^4^F_9/2_ and ^5^D_0_ energy levels of SrWO_4_: *x* Eu^3+^, 4 mol% Dy^3+^ (*x* = 0, 0.2 mol%, 0.4 mol%, 0.6 mol%, 0.8 mol%, 1 mol%) phosphors under 266 nm excitation at room temperature.

Figure 6a displays the emission curves of SrWO_4_: *x* Eu^3+^, 4 mol% Dy^3+^ (*x* = 0, 0.2 mol%, 0.4 mol%, 0.6 mol%, 0.8 mol%, 1 mol%) phosphors. The emission spectrum of the SrWO_4_: 4 mol% Dy^3+^ reveals a strong yellow (572 nm) emission and a blue (485 nm) emission corresponding to the ^4^F_9/2_ → ^6^H_13/2_ and ^4^F_9/2_ → ^6^H_15/2_ transition of Dy^3+^ ions, respectively, under the 266 nm excitation^28^. Two small emission peaks located at 660 and 750 nm are also observed, due to the transitions from ^4^F_9/2_ excited state to ^6^H_11/2_ and ^6^H_9/2_ ground states. And a very weak broad band in the range of 350–550 nm corresponding to the WO_4_^2−^ emission can be found. One can see that the hypersensitive electric dipole transition ^4^F_9/2_ → ^6^H_13/2_ at 572 nm dominates the spectrum, which indicates that the Dy^3+^ ions are placed at the sites of non-inversion symmetry^5, 29^. Four new emission peaks at 590, 615, 650 and 700 nm appear, due to the *f*-*f* transitions (^5^D_0_ → ^7^F_1,2,3,4_) of Eu^3+^ ions, along with the characteristic transitions of Dy^3+^ ^13^. The integral intensity of 572 nm and 615 nm emissions is calculated as a function of Eu^3+^ concentration as well as the total emissions, as shown in Fig. 6b. The emission intensity of Eu^3+^ (615 nm) increases with increase of the Eu^3+^ concentration from 0.2 mol% to 0.8 mol%, and then decreases when the concentration further increases above 0.8 mol% due to the concentration quenching effect^30^. The Dy^3+^ emission (572 nm) intensity increases with the increase of Eu^3+^ concentration and reaches a maximum value at Eu^3+^ concentration of 0.4 mol%, which can be ascribed to the energy transfer from Eu^3+^ to Dy^3+^ ^31^. With the continuous increasing of Eu^3+^ concentration, the Dy^3+^ emission intensity decreases, which can be attributed to the concentration quenching effect. Focusing on the total emissions intensity, when the doping concentration of Eu^3+^ reaches to 0.8 ml%, the strongest total emission intensity is obtained. Thus, the sample co-doped with 0.4 mol% Eu^3+^ and 4 mol% Dy^3+^ should be selected as the optimum doping concentration to study optical properties at different temperature.

The effective lifetimes of ^4^F_9/2_ and ^5^D_0_ energy levels can be expressed as^32^

1 $${\tau }_{eff}=\frac{\int I(t)tdt}{\int I(t)dt}$$

where *I(t)* represents the emission intensity at time *t*. The decay curves of Dy^3+^ (^4^F_9/2_) and Eu^3+^ (^5^D_0_) ions at different Eu^3+^ concentration were recorded by monitoring at 572 nm and 615 nm, respectively. The decay curves support the existence of energy transfer progress for doped and co-doped samples. The values of lifetimes of SrWO_4_: *x* Eu^3+^, 4 mol% Dy^3+^ (*x* = 0, 0.2 mol%, 0.4 mol%, 0.6 mol%, 0.8 mol%, 1 mol%) phosphors were calculated by using equation (1), in Fig. 6c. The decreasing tendency of lifetimes of both ^4^F_9/2_ and ^5^D_0_ energy levels can be found with the rise of Eu^3+^ concentration. The Fig. 6c shows the inhomogeneous change of lifetimes of the 572 nm (Dy^3+^) and 615 nm (Eu^3+^) emissions. It means that the energy transfer from charge transfer band of W-O to Dy^3+^ and the energy transfer from charge transfer band of Eu-O to Eu^3+^ as well as energy transfer from Eu^3+^ to Dy^3+^ are different at different Eu^3+^ concentrations.

**Figure 7:**
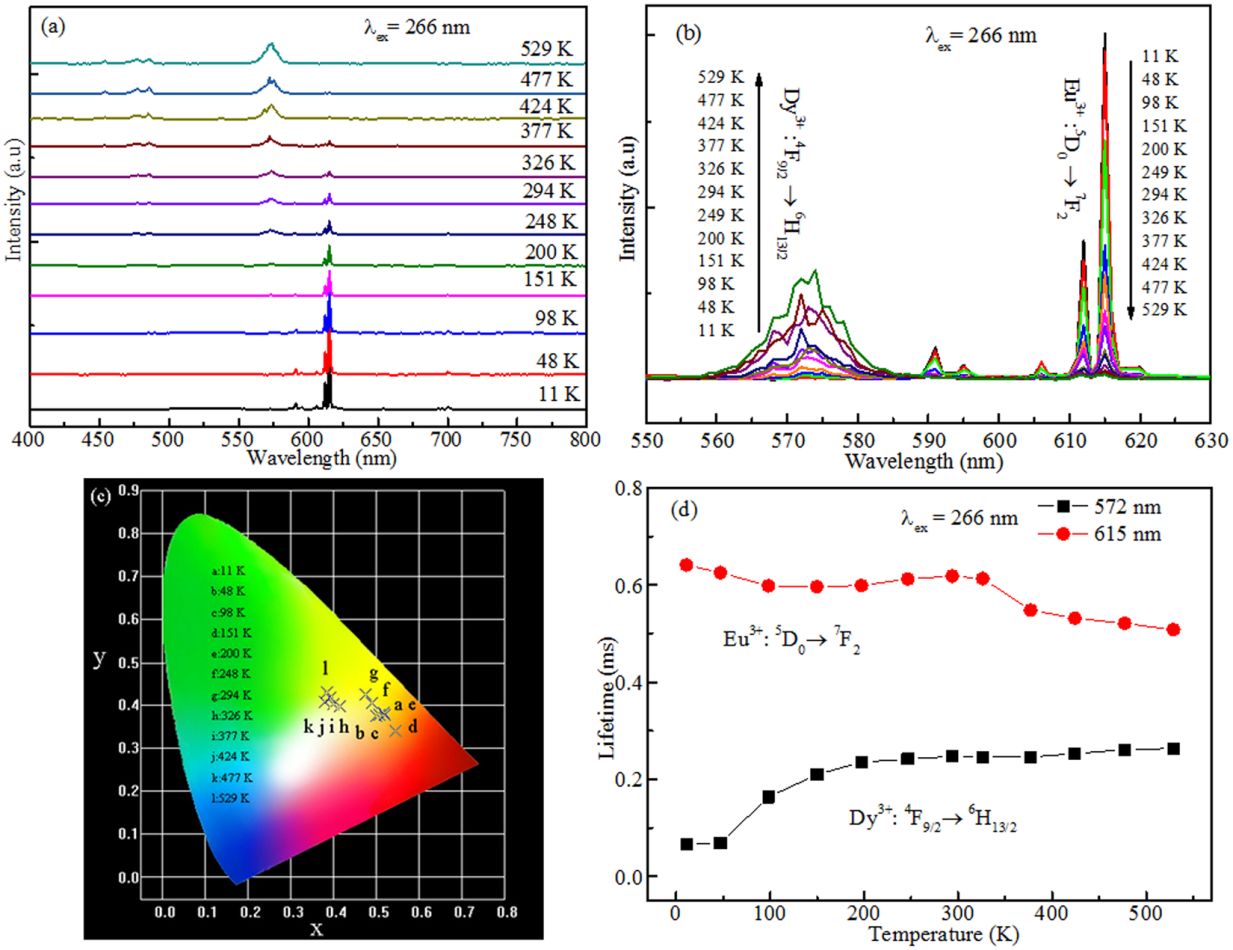
(**a**) PL Emission spectra, (**b**) Temperature-dependent spectra at 572 nm and 615 nm, (**c**) CIE, (**d**) Calculated lifetimes of ^4^F_9/2_ (Dy^3+^) and ^5^D_0_ (Eu^3+^) energy levels of Dy^3+^ and Eu^3+^ ions of the SrWO_4_: 0.4 mol% Eu^3+^, 4 mol% Dy^3+^ phosphor under 266 nm excitation from 11 K to 529 K.

To further study the temperature-dependent photoluminescence performance, the emission spectra of the SrWO_4_: 0.4 mol% Eu^3+^, 4 mol% Dy^3+^ samples are investigated in the temperature range from 11 K to 592 K, as shown in Fig. 7a. One can see that the emission intensity of Dy^3+^ ions increases with the rise of temperature, while the emission intensity of Eu^3+^ ions decreases. The emission bands of Dy^3+^ ions at 572 nm (^4^F_9/2_ → ^6^H_13/2_) and Eu^3+^ ions at 615 nm (^5^D_0_ → ^7^F_2_) were enlarged and shown in Fig. 7b. One can find that the intensity of 572 nm (Dy^3+^) increases with the temperature increase, while the intensity of 615 nm (Eu^3+^) decreases with the temperature increase. It means that the energy transfer from charge transfer bands to Eu^3+^ and Dy^3+^ ions is temperature dependent. The Commission International de L’Eclairage (CIE) diagram (Fig. 7c) shows that the emission color of the SrWO_4_: 0.4 mol% Eu^3+^, 4 mol% Dy^3+^ sample can be turned from the orange-red to the yellow region with the increase of temperature from 11 K to 529 K.

In order to study the energy transfer among charge transfer bands, Eu^3+^ and Dy^3+^, the decay curves of ^4^F_9/2_ and ^5^D_0_ energy levels at different temperature were measured by monitoring at 572 nm and 615 nm, respectively, and calculated by using equation (1). The values of the effective lifetimes are shown in Fig. 7d. It can be found that the lifetimes of ^4^F_9/2_ energy level of Dy^3+^ ion increase with the increase of temperature, while the lifetimes of ^5^D_0_ energy level of Eu^3+^ ion decrease, demonstrating the different energy transfer rates from charge transfer bands to Dy^3+^ and Eu^3+^ ions^33^.

**Figure 8:**
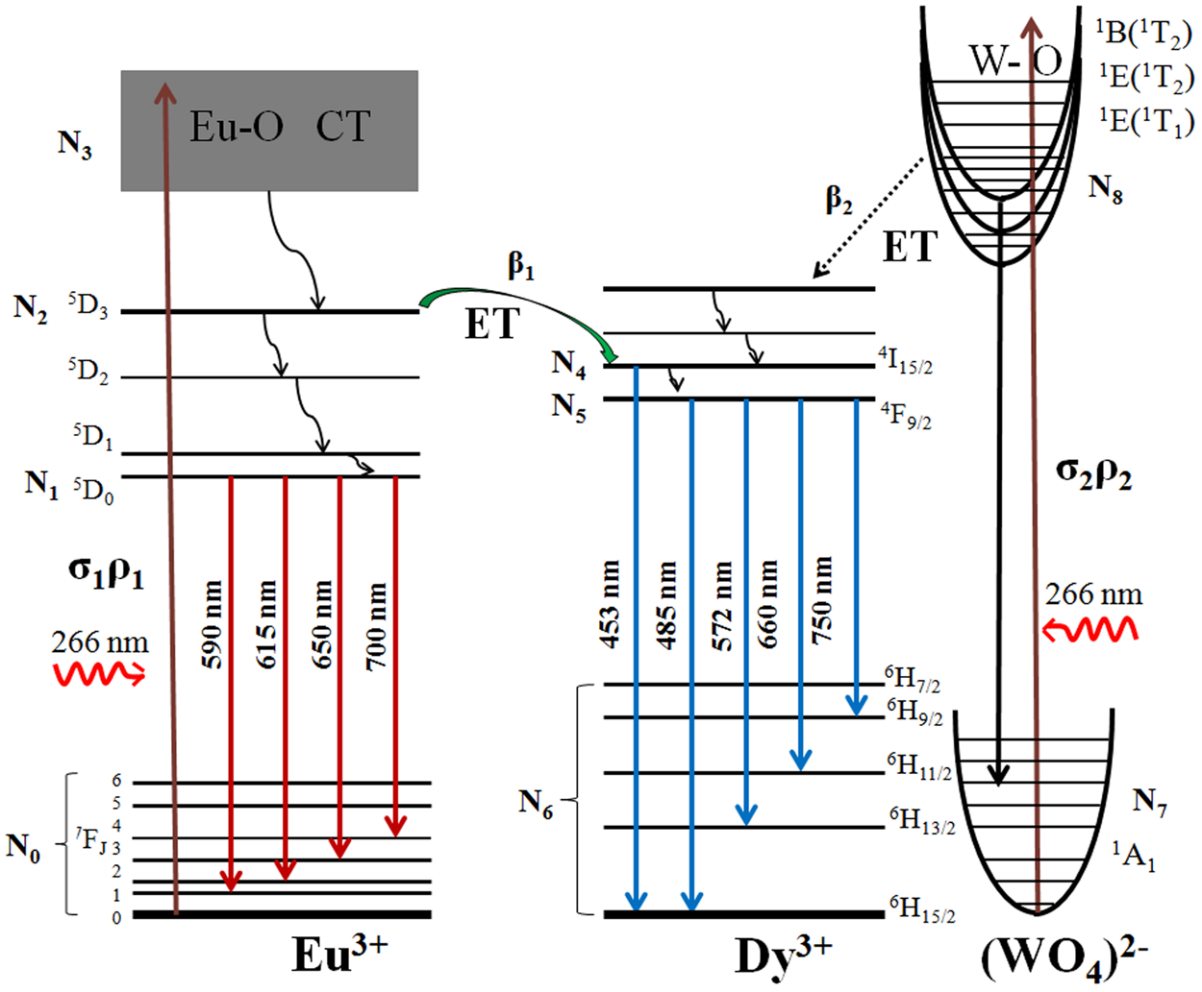
The mechanism graph of the optical temperature sensing through energy transfer from the charge transfer bands to Dy^3+^ and Eu^3+^ ions under the 266 nm excitation.

To study the temperature dependence of energy transfer from charge transfer bands to Eu^3+^-Dy^3+^ ions, the dynamic balance rate-equation model for the energy transfer between charge transfer bands and Eu^3+^-Dy^3+^ ions are established in Fig. 8. We supposed ^7^F_J_(J = 0, 1, 2, 3, 4, 5, 6), ^6^H_J/2_ (J = 15, 13, 11, 9, 7), or ^1^B(^1^T_2_)/^1^E(^1^T_2_)/^1^E(^1^T_1_) energy levels as a same level in the case of the fixed temperature. The energy transfer between Eu^3+^ and WO_4_^2-^ is neglected. The corresponding rate equations are as follows:

2 $$\frac{d{N}_{1}}{dt}={N}_{2}{W}_{21}-{N}_{1}{A}_{10}$$

3 $$\frac{d{N}_{2}}{dt}={N}_{3}{W}_{32}-{\beta }_{1}{N}_{2}{N}_{4}-{N}_{2}{W}_{21}$$

4 $$\frac{d{N}_{3}}{dt}={\sigma }_{1}{\rho }_{1}N{}_{0}-{N}_{3}{W}_{32}$$

5 $$\frac{d{N}_{5}}{dt}={N}_{4}{W}_{45}-{N}_{5}{A}_{56}$$

6 $$\frac{d{N}_{8}}{dt}={\sigma }_{2}{\rho }_{2}{N}_{7}-{N}_{8}{A}_{87}-{\beta }_{2}{N}_{8}{N}_{4}$$

where *σ*_1_ and *σ*_2_ are the cross-section of the ground state absorption of ^7^F_J_ and ^1^A_1_, *ρ*_1_ and *ρ*_2_ are the incident pumping power density, *N*_0_, *N*_1_, *N*_2_, *N*_3_, *N*_4_, *N*_5_, *N*_6_, *N*_7_, and *N*_8_ are the population densities of the levels of Eu^3+^, (WO_4_)^2−^, and Dy^3+^ respectively. β_1_ and β_2_ correspond to the energy transfer rates from ^5^D_3_ and ^1^B(^1^T_2_)/^1^E(^1^T_2_)/^1^E(^1^T_1_) to ^4^I_15/2_, respectively. The terms of *W*_*ij*_ represent the nonradiative decay rates between the levels *i* and *j*, *A*_*ij*_ is the radiative transition rates between the levels *i* and *j*.

By solving the above equations, we have

7 $$\frac{{N}_{5}}{{N}_{1}}\approx \frac{{W}_{45}{A}_{10}}{{A}_{56}{W}_{21}{\sigma }_{1}{\rho }_{1}{N}_{0}}(\frac{{W}_{21}}{{\beta }_{2}}+\frac{{\beta }_{1}}{{\beta }_{2}^{2}})$$

The nonradiative relaxation possibility is proportional to ref. 34

8 $${w}_{ij}\propto {e}^{-\hslash w/kT}$$

The luminescence intensity of an emission band can be expressed as

9 $${I}_{ij}=h{v}_{i}{A}_{ij}{N}_{i}$$

where *hν*_*i*_ is transition energy per photon, *A*_*ij*_ is spontaneous radiative emission probability from an *i* state to a *j* state, and *N*_*i*_ is the state population of the *i* state^35^.

The emission intensity ratio of Dy^3+^ (572 nm) and Eu^3+^ (615 nm) ions, defined as *FIR* (I_Dy_/I_Eu_), is adopted to study the temperature-dependent photoluminescence property. Combining with above equations, the *FIR* (I_Dy_/I_Eu_) can be fitted as

10 $$FIR=\frac{{I}_{Dy}}{{I}_{Eu}}=\frac{A\exp (-2\hslash \omega /kT)}{1-\exp (-\hslash \omega /kT)}+B$$

where *A* is the fitting constant that depends on the experimental system and intrinsic spectroscopic parameter; *ħω* is the phonon energy; and *k* is a Boltzmann constant^36^. The absolute sensitivity and relative sensitivity can be defined as^37^

11 $${S}_{a}=\frac{dR}{dT}=\frac{{\rm{A}}\hslash \omega \exp (-3\hslash \omega /kT)}{k{(1-\exp (-\hslash \omega /kT))}^{2}{T}^{2}}+\frac{{\rm{2A}}\hslash \omega \exp (-2\hslash \omega /kT)}{k(1-\exp (-\hslash \omega /kT)){T}^{2}}$$

12 $${S}_{r}=\frac{1}{R}\frac{dR}{dT}=\frac{\frac{{\rm{A}}\hslash \omega \exp (-3\hslash \omega /kT)}{k{(1-\exp (-\hslash \omega /kT))}^{2}{T}^{2}}+\frac{{\rm{2A}}\hslash \omega \exp (-2\hslash \omega /kT)}{k(1-\exp (-\hslash \omega /kT)){T}^{2}}}{B+\frac{A\exp (-2\hslash \omega /kT)}{1-\exp (-\hslash \omega /kT)}}$$

**Figure 9:**
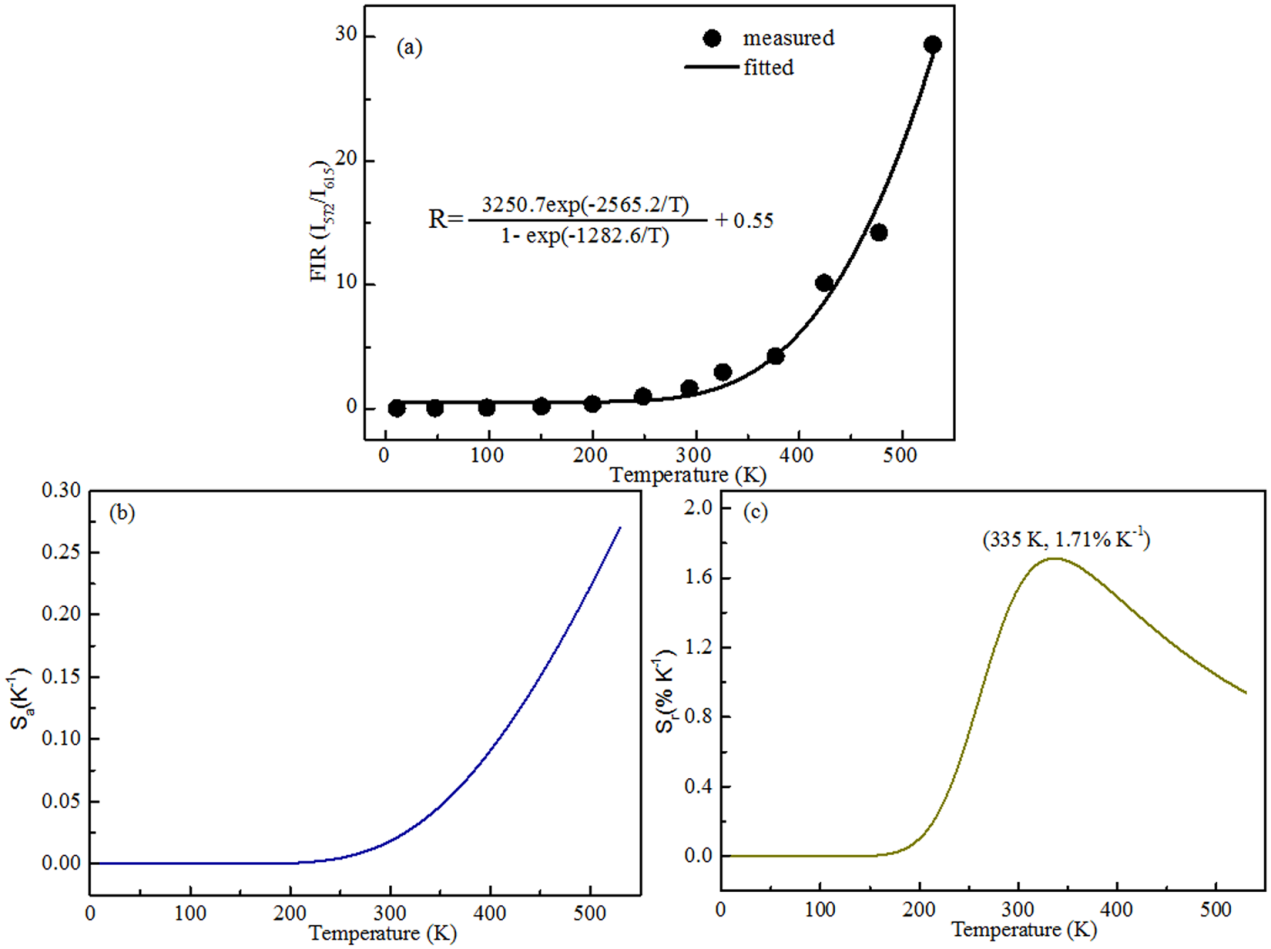
(**a**) Experimental measured and fitted plots of *FIR* (I_572_/I_615_) versus temperature. (**b**) Absolute sensitivity S_a_ and (**c**) Relative sensitivity S_r_ versus temperature.

As displayed in Fig. 9a, the *FIR* data could be exponentially fitted by the equation (10) from 11 K to 529 K. The parameters *A*, *B* and *ħω* can be determined to be 3250.7, 0.55 and 903.8 cm^−1^ for the SrWO_4_: 0.4 mol% Eu^3+^, 4 mol% Dy^3+^ sample by using the fitting equation. The fitted phonon energy of 903.8 cm^−1^ is closed to the literature reported of 917.7 cm^−1^ ^38^. The error of the fitted phonon energy is about 1.5%. On the basis of the equations (11) and (12), the absolute sensitivity S_a_ and relative sensitivity S_r_ are calculated and shown in Fig. 9b,c. One can see that the absolute sensitivity is as high as 0.27 K^−1^ at 529 K. It is much higher than the literature reported^39, 40^. For example, the absolute sensitivity in Eu^3+^ doped Gd_2_Ti_2_O_7_ phosphor was 0.015 K^−1^ ^41^, and in Dy^3+^ doped GdVO_4_ phosphor was 0.01 K^−1^ ^42^. The maximum relative sensitivity of 1.71% K^−1^ is obtained at 335 K. It is higher than the reported phosphors, 0.014 K^−1^ in Eu^3+^ doped CaGd_2_(WO_4_)_4_ scheelite^43^ and 0.003 °C^−1^ in Dy^3+^ doped Y_4_Al_2_O_9_ phosphor^44^. The improvement of both the relative sensitivity and absolute sensitivity of this material may be owing to different energy transfer ratio from charge transfer bands to Eu^3+^-Dy^3+^ ions at different temperatures, leading to a significant change in the emission intensity ratio.

**Figure 10:**
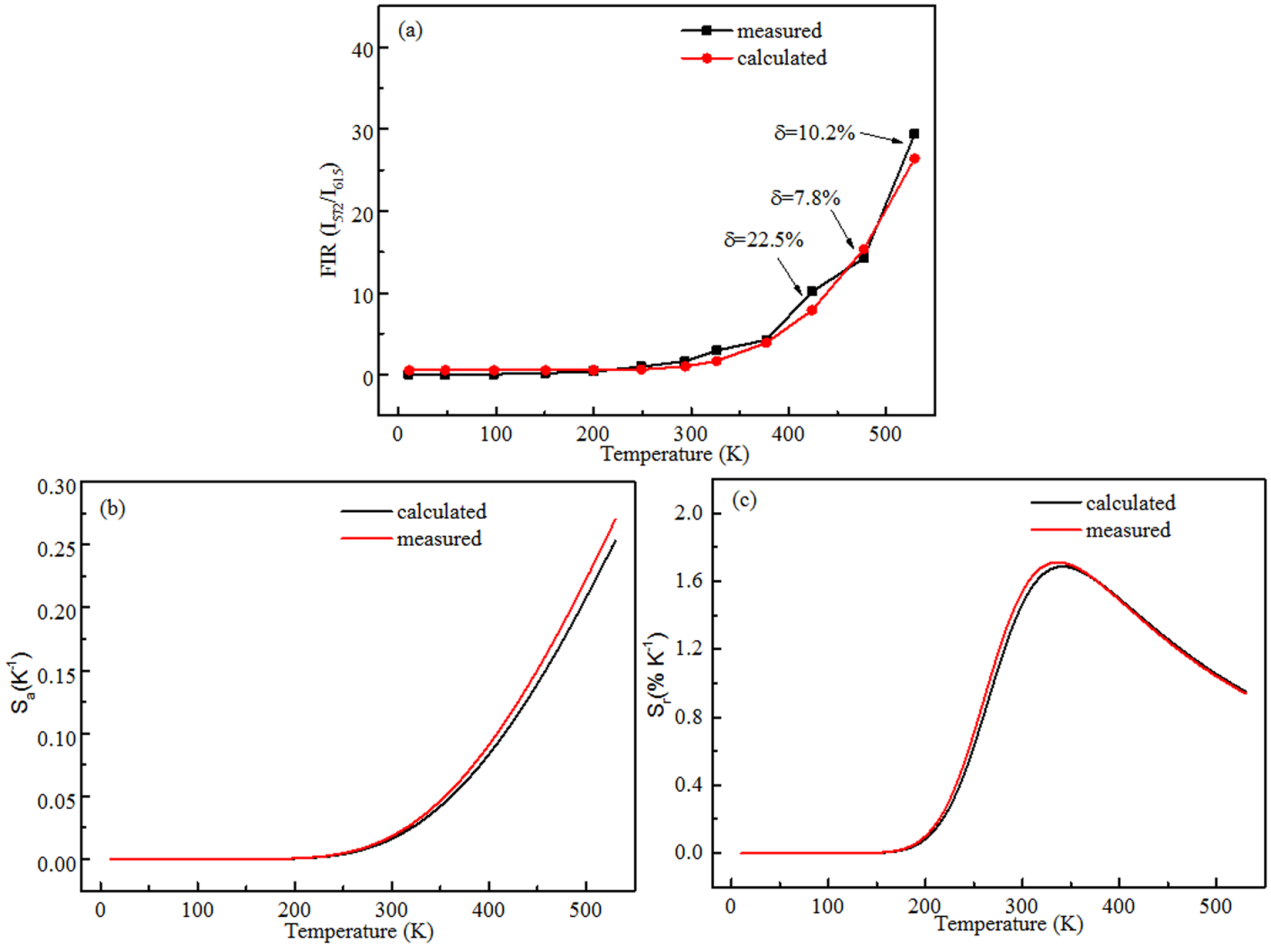
(**a**) Experimental measured and calculated plots of *FIR* (I_572_/I_615_) versus temperature. (**b**) Measured and calculated absolute sensitivity S_a_ and (**c**) relative sensitivity S_r_ versus temperature.

The error analysis of measured and calculated *FIR*(I_572_/I_615_) is shown in Fig. 10a. One can see that the measured and the calculated *FIR* match well at low temperature, while the error appears at high temperature more than 400 K. The error may originated from the active nonradiative relaxation and energy transfer between Eu^3+^/Dy^3+^ ions and host^39, 45^. Notably, this error affects little on the values of S_a_ and S_r_, as shown in Fig. 10b,c.

## Conclusions

In this work, a series of Eu^3+^/Dy^3+^ co-doped SrWO_4_ phosphors were prepared by the high-temperature solid-state method. The structural property was studied by the X-Ray diffraction. The emission intensity, fluorescence color, and lifetimes of Dy^3+^ (572 nm) and Eu^3+^ (615 nm) of the SrWO_4_: 0.4 mol% Eu^3+^, 4 mol% Dy^3+^ are investigated in the temperature range from 11 K to 529 K under the 266 nm excitation. The emission intensity ratio of Dy^3+^ and Eu^3+^ ions was found to be temperature dependent. The maximum value of S_r_ can be reached 1.71% K^−1^ at 335 K, being higher than those previously reported material. This work opens a new route to obtain optical thermometry with high sensitivity through using down-conversion fluorescence under ultraviolet excitation.

## Methods

A series of Eu^3+^/Dy^3+^ single doped and co-doped SrWO_4_ phosphors were prepared by the high-temperature solid-state method. According to the appropriate stoichiometric ratio, the starting materials, SrCO_3_ (Aldrich, 99.9%), WO_3_ (Aldrich, 99.9%), Eu_2_O_3_ (Aladdin, 99.99%), and Dy_2_O_3_ (Aldrich, 99.99%) were weighted and ground thoroughly in an agate mortar for 30 minutes with ethanol. Then the homogenous mixture was collected into a crucible and sintered at 1000 °C for 4 hours. After cooling to the room temperature, the obtained white samples were ground to powder for further investigation.

The obtained products were characterized by X-ray diffraction (XRD) using a Philips X’Pert MPD (Philips, Netherlands) X-ray diffractometer at 40 kV and 30 mA. All patterns are recorded in the range of 10–90° with a step size of Δ2*θ* = 0.02. The morphology, particle size and energy dispersive spectrometer (EDS) of the phosphor are characterized by scanning electron microscope (SEM) system (JSM-6490, JEOL Company). The ultraviolet-visible diffuse reflectance spectrum is recorded using a V-670 (JASCO) UV-vis spectrophotometer. The photoluminescence excitation (PLE) spectra are recorded by a Pjoton Technology International (PTI, USA) fluorimeter with a 60 W Xe-arc lamp as the excitation light source at room temperature. The photoluminescence (PL) spectra and decay lifetimes are collected by a 266 nm-pulsed laser with a pulse width of 5 ns and a repetition rate of 10 Hz (Spectron Laser Sys. SL802G).
